# Local Vibration Reduces Muscle Damage after Prolonged Exercise in Men

**DOI:** 10.3390/jcm10225461

**Published:** 2021-11-22

**Authors:** Anna Piotrowska, Wanda Pilch, Łukasz Tota, Marcin Maciejczyk, Dariusz Mucha, Monika Bigosińska, Przemysław Bujas, Szczepan Wiecha, Ewa Sadowska-Krępa, Tomasz Pałka

**Affiliations:** 1 Institute of Basics Sciences, Faculty of Physiotherapy, University of Physical Education, 31-571 Kraków, Poland; anna.piotrowska@awf.krakow.pl (A.P.); wanda.pilch@awf.krakow.pl (W.P.); 2 Department of Physiology and Biochemistry, Faculty of Physical Education and Sport, University of Physical Education, 31-571 Kraków, Poland; lukasz.tota@awf.krakow.pl (Ł.T.); tomasz.palka@awf.krakow.pl (T.P.); 3 Institute of Biomedical Sciences, Faculty of Physical Education and Sport, University of Physical Education, 31-571 Kraków, Poland; dariusz.mucha@awf.krakow.pl; 4 Department of Physical Education, Institute of Physical Culture, State University of Applied Sciences, 33-300 Nowy Sącz, Poland; mbigosin@poczta.onet.pl; 5 Institute of Sports, University of Physical Education, 31-571 Kraków, Poland; przemyslaw.bujas@awf.krakow.pl; 6 Department of Physical Education and Health, Faculty in Biala Podlaska, Jozef Pilsudski University of Physical Education in Warsaw, 21-500 Biala Podlaska, Poland; szczepan.wiecha@awf-bp.edu.pl; 7 Institute of Sport Sciences, The Jerzy Kukuczka Academy of Physical Education, 40-065 Katowice, Poland; e.sadowska-krepa@awf.katowice.pl

**Keywords:** DOMS, vibration, post-exercise muscle damage, physical exercise, recovery

## Abstract

Prolonged exercise can lead to muscle damage, with soreness, swelling, and ultimately reduced strength as a consequence. It has been shown that whole-body vibration (WBV) improves recovery by reducing the levels of stress hormones and the activities of creatine kinase (CK) and lactate dehydrogenase (LDH). The aim of the study was to demonstrate the effect of local vibration treatment applied after exercise on the level of selected markers of muscle fiber damage. The study involved 12 untrained men, aged 21.7 ± 1.05 years, with a VO_2_peak of 46.12 ± 3.67 mL·kg^−1^·min^−1^. A maximal intensity test to volitional exhaustion was performed to determine VO_2_peak and individual exercise loads for prolonged exercise. The subjects were to perform 180 min of physical effort with an intensity of 50 ± 2% VO_2_peak. After exercise, they underwent a 60 min vibration treatment or placebo therapy using a mattress. Blood samples were taken before, immediately after the recovery procedure, and 24 h after the end of the exercise test. Myoglobin (Mb) levels as well as the activities of CK and LDH were recorded. Immediately after the hour-long recovery procedure (vibration or placebo), the mean concentrations of the determined indices were significantly different from baseline values. In the vibration group, significantly lower values of Mb (*p* = 0.005), CK (*p* = 0.030), and LDH (*p* = 0.005) were seen. Differences were also present 24 h after the end of the exercise test. The results of the vibration group compared to the control group differed in respect to Mb (*p* = 0.002), CK (*p* = 0.029), and LDH (*p* = 0.014). After prolonged physical effort, topical vibration improved post-workout recovery manifested by lower CK and LDH activity and lower Mb concentration compared to a control group.

## 1. Introduction

Prolonged strenuous exercise can lead to muscle cell damage, with soreness, swelling, and ultimately a decrease in strength as a consequence, especially when the athlete is not used to this type of physical work or performs it after a long absence [1]. The damages take place on the ultrastructure of muscle cells. It has been reported that eccentric contractions [2] and forms of exercise unfamiliar to the body most often contribute to its damage. Clinical symptoms include decreased strength, painful restricted range of movement, stiffness, swelling, and dysfunction of adjacent joints, one of the most common causes of decreased performance in professional athletes. The development of clinical symptoms is usually delayed, occurring between 8 to 20 h after exercise and, as a result of the complex sequences of local and systemic physiological responses, may persist for 24 to 48 h after the injury has occurred [1,3]. This comprehensive picture of subjective and objective symptoms is defined as Delayed Onset Muscle Soreness (DOMS). Among the physiological and biochemical changes, the development of an inflammatory state is followed by an increase in the activity of cellular enzymes and inflammatory metabolites in the blood, a loss of muscle strength, and a decline in endurance [1,4].

Currently, there is growing interest in new methods of biological regeneration, aimed to accelerate the pace of recovery and reduce post-exercise skeletal muscle damage [5]. Scientific reports have mostly evaluated the use of systemic cryotherapy [6] and massage [7]. However, there are also studies evaluating various forms of vibration. Different protocols have incorporated the use of vibration either before, during breaks between efforts, or after the physical performance. Preliminary meta-analyses have shown promising results while also emphasizing the need for further research on this topic [8].

Vibration as a stimulus that positively affects health has been known for a long time, but the exact mechanism and effectiveness are yet to be understood [9]. The largest scientific study used cold whole-body vibration (WBV). The stimulus was applied in a standing position through the feet. WBV has been demonstrated to improve muscle perfusion and improve lymph flow. It significantly affects the supply of oxygen and other components important for the working muscle [10,11,12]. Recently, WBV has also been evaluated as a post-exercise recovery treatment to reduce muscle pain and loss of muscle strength after exercise [13,14]. Apart from subjective indicators, a decrease in stress hormones [15] and creatine kinase (CK) activity [16] has been observed. Vibration as a mechanical stimulus used in the form of WBV is absorbed mainly by the skeletal system.

The local application of vibration in wellness has been evaluated in a few studies. In addition to WBV, its local use is another form of stimulus propagation that causes smaller changes in the skeletal system. However, the reaction of the soft tissues to this stimulus is strong, as shown in biochemical studies [17] and thermal imaging [18]. Locally applied vibration contributes to the development of neuromuscular adaptations after both single and multiple uses [19]. Vibration applied locally will be absorbed by the soft tissues and will have the greatest impact on them. Therefore, we expect the effect to be equally effective despite the use of a gentler local vibration stimulus. 

Changes in the levels of biochemical indicators can assess the degree of damage a muscle cell endures after prolonged physical exertion. Therefore, a research question was posed: will the use of local vibration affect specific indices present immediately after physical exercise and increase the rate of post-exercise recovery by reducing the concentration of post-exercise biochemical indices of muscle cell damage? Our hypothesis assumed that vibration treatment, thanks to increased vascular flow and improved tissue drainage, would reduce the blood markers of muscle damage.

## 2. Materials and Methods

### 2.1. Ethics Declaration

The research was carried out in the Laboratory of Vibrotherapy and the Laboratory of Physiological Basis of Adaptation, Central Scientific and Research Laboratory, University of Physical Education in Krakow during September–October 2019. The respondents gave written consent to participate in the research, were explained the purpose and method of the research, and were informed about the possibility of resignation at any stage without giving a reason. The project was approved by the Bioethical Committee of PMWSZ in Opole (No. KB/56/N02/2019) in accordance with the Declaration of Helsinki. The proposed intervention in the form of vibrotherapy met the guidelines for reporting research using whole-body vibration as a treatment regimen in humans [20,21].

### 2.2. Study Protocol

**Figure 1:**
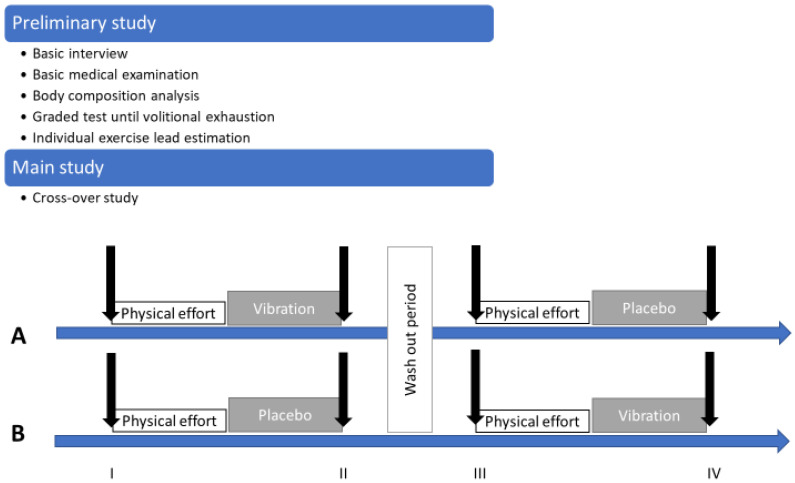
Study protocol with preliminary and main study details. (**A**,**B**) are the names of the groups taking part in the cross-over study. Black arrows represent the instants of time that blood samples were taken. Intervention type: vibration or placebo. I—first blood sampling (before physical effort); II—second blood sampling (1 h after physical effort); III—third blood sampling (before second physical effort); IV—fourth blood sampling (1 h after second physical effort).

The protocol included two series of studies: a preliminary one and a main one (Figure 1). In the preliminary study, a basic interview and basic medical examination were performed, and body composition was estimated with the use of an analyzer. At this stage, a graded test until volitional exhaustion was performed during which selected physiological indicators were measured. The obtained values of the graded tests made it possible to estimate the individual exercise load at a level of 50 ± 2% VO_2_peak necessary for the implementation of the pivotal test.

Our study included 12 untrained but physically active male students from the University of Physical Education in Krakow who met the inclusion criteria, and in the medical examination, there were no contraindications to the use of vibrotherapy (Table 1).

In the main study (experimental intermittent study), men were randomly divided into two 6-person groups: A and B. All examined men performed a physical effort with individual estimated load. After exercise, the participants were subjected to vibration treatments or a placebo treatment. In the first part, group A received vibrotherapy treatments after exercise while group B served as the control group by resting on placebo mattresses after exercise. After two weeks, the protocols were changed and the participants from group A received treatment on placebo mattresses, while group B received vibration treatments. Blood was collected before (0), immediately after the renewal procedure (I), and 24 h (II) after the end of the exercise test (Figure 1).

### 2.3. Study Group

The participants’ baseline characteristics (mean ± SD) were an age of 21.7 ± 1.05 years, a body height (BH) of 179.3 ± 4.3 cm, a body mass (BM) of 76.01 ± 3.4 kg, a percentage of body fat (BFP) of 13.72 ± 2.7%, a lean body mass (LBM) of 65.56 ± 2.97 kg, a Body Mass Index (BMI) of 23.64 ± 0.54, and a VO_2_peak of 46.12 ± 3.67 mL·kg^−1^·min^−1^. The values of the physiological indices monitored during the exercise test until exhaustion are presented in Table 2. During the entire research project, the subjects were advised not to change their current diet and refrain from consuming alcohol and taking up additional forms of physical activity.

### 2.4. Data Collection

The body compositions (BH, BM, BMI, BFP, LBM) were estimated with the use of an analyzer (JAWON MEDICAL IOI-353-CE0197-Korea certificate). A graded test until volitional exhaustion [23] was performed on a cycloergometer during which maximal heart rate (HRmax), peak oxygen consumption (VO_2_peak), maximal pulmonary ventilation (VEmax), maximal breathing frequency (FRmax), maximal tidal volume (TVmax) and the amount of total work performed (TW) were measured. During the test, the levels of cardiopulmonary indices were recorded based on the “breath-by-breath” method using an ergospirometer. Data were averaged every 30 s. The highest registered value of oxygen uptake was considered as peak oxygen uptake. The obtained maximum values of the graded tests made it possible to estimate the individual exercise load at a level of 50 ± 2% VO_2_peak necessary for the implementation of the pivotal test.

The following measuring devices were used: cycloergometer (ER 900D, Jaeger, Hoechberg, Germany), ergospirometer (MetaLyzer, Cortex, Leipzig, Germany), and cardiomonitor (RS 400, Polar Elektro, Kempele, Finland). The ergospirometer was calibrated in accordance with the manufacturer’s recommendations (gas and volume calibration). All physiological tests at this stage were carried out in the morning in the air-conditioned Laboratory of Physiological Basis of Adaptation of the University of Physical Education in Krakow (PN-EN ISO 9001: 2015).

### 2.5. Biochemical Analysis

Blood was collected from the antecubital vein in a volume of 15 mL in accordance with the applicable standards of the laboratory diagnostician before (0), immediately after the renewal procedure (I), and 24 h (II) after the end of the exercise test.

Myoglobin (Mb), CK, and LDH determinations were performed using the enzyme immunoassay technique—ELISA, using the E-LizaMat3000 apparatus (DRG Instruments Gmbh, Marburg, Germany) with use of kits: Mb: EIA-3955 (DRG GmbH, Marburg, Germany), sensitivity (the lowest detectable level: 5 ng/mL); CK: EIA-4361 (DRG GmbH, Marburg, Germany), sensitivity (the lowest detectable level: 0.5 ng/mL); LDH: MAK066 (Sigma-Aldrich Co., St. Louis, MO, USA). To determine changes in plasma volumes of the blood samples, the following was determined: hemoglobin concentrations using the Drabkin method and hematocrit levels using the micro-hematocrit method. Changes in plasma volume (%ΔPV) were calculated using the formula of Dill and Costila [24] modified by Harisson et al. [25] The concentrations of biochemical markers in the blood samples collected after exercise were corrected for %∆PV.

### 2.6. Intervention

All examined men performed 180 min of physical effort on cycloergometer at a relative intensity of 50 ± 2% VO_2_peak. A 10 min warm-up using the same intensity was performed prior to beginning the test. Immediately after exercise, the body was dried and BM was re-measured followed by a four-minute shower (water temperature: 21 ± 2 °C).

**Figure 2:**
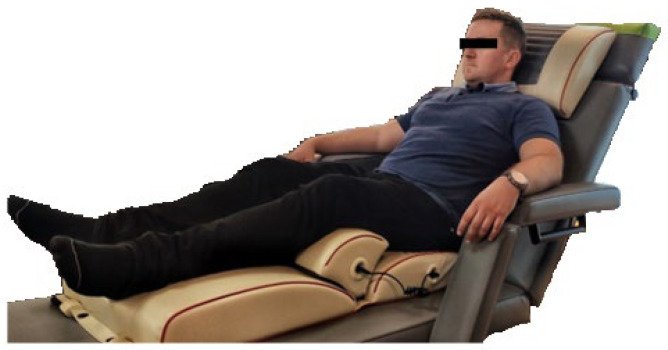
The position of the body on the device generating the vibration stimulus (illustrative photo).

**Figure 3:**
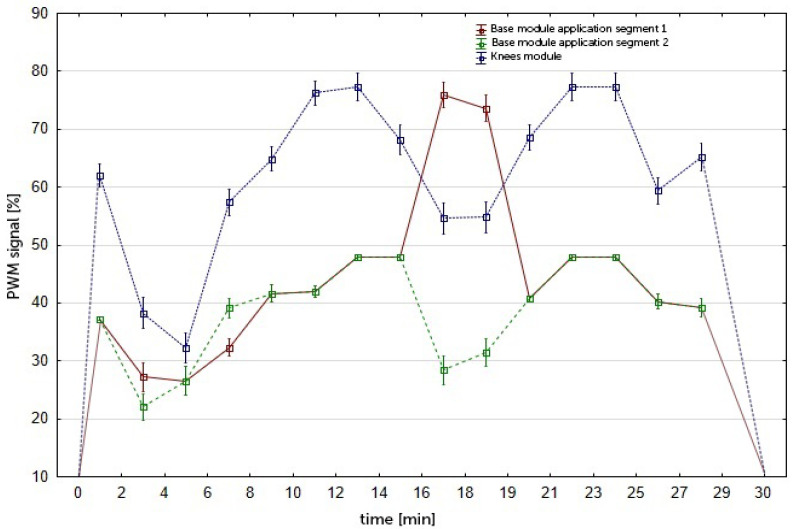
Voltage-current characteristics of the Knees program with the Knees S module (PWM: Pulse Width Modulation).

The participants in the vibration group then underwent a 60 min lower body vibration massage in a reclining position using a RAM Vitberg+ Massage Device (Vitberg, Nowy Sącz, Poland) (Figure 2. The duration of the treatment was determined according to the manufacturer’s information and consisted of two 30 min cycles. The therapeutic stimulus generated by the device was cycloidal vibrations directed in three perpendicular directions, with a small amplitude, a low to medium frequency, and a variable sequence of impulses (f = 20–52 Hz, A = 0.1–0.5 mm, a = 6.9–13.5 m/s^2^). During the 60 min treatment, the vibrations were interrupted at different values of frequency, amplitude, and acceleration (Figure 3). The device has a TUV Rheinland certificate (No. 0197) and a quality certificate for class IIa medical devices (No. HD60118119001). 

Placebo treatments were carried out on specially designed Vitberg+ placebo devices, which in terms of shape, appearance, and equipment were identical to the active ones. They generated the same sound signals during individual phases of the placebo treatment but did not produce the vibrations tested in the experimental group.

### 2.7. Statistical Analysis

The test results for groups A and B using the vibrating devices and placebo devices were blinded, and the following groups were identified as vibrated (V) and placebo (C). All results were presented as arithmetic means with standard deviations. In order to assess the significance of differences between groups, and time changes, analysis of variance (ANOVA) with repeated measurements was used. Afterwards, post hoc analysis was carried out using Tukey’s test. Data distribution was checked using the Shapiro–Wilk test. Homogeneity of variance within the groups was tested via Levene’s test (variance of the analyzed parameters was similar in both groups). The effect size (partial eta squared (η^2^)) was calculated and interpreted as small (0.01), medium (0.06), or large (0.14) [26]. Additionally, in post hoc analysis, the effect size (d-Cohen) between groups was calculated and interpreted as small (0.20), medium (0.50), or large (0.80) [26]. Observed statistical power (post hoc power) is also reported. Statistically significant results were defined as a *p*-value of <0.05. The following software was used to perform the calculations: STATISTICA 13.1 (StatSoft, Tulsa, OK, USA).

## 3. Results

In the study, significant time changes were noted for LDH (f = 156.7, *p* < 0.001, η^2^ = 0.87, observed power: 1.0), CK (f = 3427.7, *p* < 0.001, η^2^ = 0.99, observed power: 1.0), and Mb (f = 20.46, *p* < 0.001, η^2^ = 0.48, observed power: 0.99). The studied groups differ with regard to the examined indices (LDH: f = 4.5, *p* = 0.04, η^2^ = 0.17, observed power: 0.47; CK: f = 4.56, *p* = 0.049, η^2^ = 0.17, observed power: 0.47; Mb: f = 4.61, *p* = 0.04, η^2^ = 0.18, observed power: 0.26). Moreover, there were significant interactions between the analyzed factors (group × time): (LDH: f = 15.02, *p* < 0.001, η^2^ = 0.41, observed power: 0.99; CK: f = 8.06, *p* = 0.001, η^2^ = 0.27, observed power: 0.94; Mb: f = 4.56, *p* = 0.04, η^2^ = 0.25, observed power 0.48).

Post-hoc analysis indicated that the concentrations of Mb and activities of CK and LDH in the C and V groups taken before exercise (0) did not differ significantly. Immediately after an hour-long recovery procedure (vibration or placebo) (I), the mean concentrations of the indices increased significantly in relation to the baseline in both groups. All values differed between C and V groups. Similar differences were seen in the results obtained 24 h after the end of the exercise test (II). Thus, the average values of Mb concentrations and activities of CK and LDH in the V group were significantly lower than in the C group. After 24 h from the end of the exercise test, a significant decrease in Mb concentrations and LDH activity was observed in group V while the CK activity was still increasing but was significantly lower than in the control group. Twenty-four hours after the exercise, the Mb concentration in the vibration group was similar to baseline measurement (Table 3).

The effectiveness of the vibration treatments in accelerating recovery after prolonged physical exercise was assessed by analyzing the dynamic changes in concentrations of biochemical indicators (Δ) recorded immediately after the renewal procedure (I) and after 24 h of restitution (II). The average value of the increases/decreases of the analyzed variables (ΔMb, ΔLDH, and ΔCK) in men subjected to vibrations differed significantly compared to the control group (Table 4).

## 4. Discussion

In our study, we indicated that the use of vibration treatments after prolonged exercise may reduce the increase in indicators of post-exercise muscle damage. The observed lower concentrations and activities of markers reflecting the degree of muscle fiber damage after exercise in subjects receiving vibrotherapy compared to placebo procedures constitutes important evidence of the effectiveness of post-exercise vibration treatments in recovery after prolonged exercise. In our opinion, this effect may translate into a reduction in DOMS symptoms.

The cycloidal vibrations with variable parameters of intensity, amplitude, and frequency (Figure 2) produced significant reductions in CK and LDH activities and Mb concentrations compared to the control group. The comparison of results obtained in the control group made it possible to identify differences in the values of the tested parameters. The selected time points (after 1 h of recovery and after 24 h from the end of exercise) illustrated that the selected physical effort caused an increase in the examined parameters (I). After 24 h, Mb and LDH had already decreased in both groups. For CK, the time of 24 h was too short, and the activity of this enzyme continued to increase. Such changes are characteristic of strain injuries, as previously observed [27,28]. However, the proposed protocol using vibratory therapy allowed the capturing of significant differences.

As indicated in the introduction, the effectiveness of vibration in sports has not been fully established. The available data indicate that vibration therapy can be used preventively as well as therapeutically after physical effort [29]. Vibrations increase neuromuscular activity by modulating proprioceptive function and increase muscle strength [8,29]. Vibrotherapy has effects on hormone levels and lymphatic drainage, leading to a reduction in pain and improvement in mood [30]. The mechanism for pain relief, one of the primary symptoms of DOMS, involves the activation of large diameter fibers while suppressing the transfer activity of small diameter fibers [31,32]. The mechanism of action in relieving other symptoms of DOMS can be drawn from the work published by Weerakkody et al. [33] The authors concluded that vibration results in the stimulation of large diameter afferent nerve fibers within a muscle which block the neurological “gate” in the spinal cord, preventing the symptoms of DOMS from being transmitted to the brain. In addition, they carried out tests at various frequencies which allowed for the selection of the optimal parameter (80 Hz).

In additional studies, the role of vibratory stimuli in reducing the symptoms of DOMS has been expanded upon. Koeda et al. [34] demonstrated the effectiveness of vibrations applied to the elbow flexor muscles. In addition to the beneficial but subjective effects observed by the authors of this work, vibration resulted in an increase in range of motion and blood flow. The latter effect is illustrated by an increase in skin temperature after applying a vibratory stimulus [18]. However, this relationship is not so obvious. In the study of Moreira-Marconi et al. [35], when using a 60 s exposure to WBV, the skin temperature of the posterior lower limbs decreased. The authors associated this finding with a redistribution of blood to working muscles. This is another difference between the action of local vibrations, as in our study, and WBV.

Vibration has been shown to significantly reduce the amount of interleukin-6 released and the influx of morphotic blood components involved in the inflammatory response [36,37]. All these mechanisms allow one to control the severity and extent of inflammation caused by prolonged exercise.

A study concerning the effect of vibrotherapy on muscle soreness and CK activity after eccentric exercise was conducted by Bakhitiary et al. [38]. The main difference between the protocols used by Bakhitiary et al. and us was in the timing of when the vibration was applied. In our work, it was used post-workout. Bakhitiary et al. randomized 50 untrained volunteers into two equal groups: one received vibrotherapy and one received no therapy. Vibratory stimuli (50 Hz) were applied locally to the left and right quadriceps, thigh, and calf muscles for 1 min. Then, both groups performed physical exercise with a predominance of eccentric contractions on the treadmill at a speed of 4 km/h. After 24 h, serum CK activity and the level of subjective pain directly related to the DOMS were measured. The results showed a significantly increased mean value of DOMS symptoms and CK activity in the non-vibration group as compared to the vibrated group. The authors of the study indicated that this proves that vibration applied before exercise can prevent and control DOMS. Similarly, in the paper by Imtiyaz et al. [39], the authors tried to determine how vibration and massage affect not the alleviation of DOMS-related ailments but also its prevention. Two experimental groups received vibration therapy (50 Hz vibration for five minutes) or manual massage (15 min) just before exercise with a predominance of eccentric contractions. The control group did not receive any treatment. The following characteristics were assessed to document changes in the muscle’s condition: muscle soreness (pain sensation), range of motion, maximum isometric force (MIF), one-repetition maximum, and the selected biochemical indices, LDH and CK. The results of the study showed that muscle pain was significantly less in the experimental groups (vibration and massage) compared to the control group at 24, 48, and 72 h after exercise. The experimental and control group showed no significant differences in MIF. The use of vibration therapy resulted in significantly lower LDH and CK activities at 48 h after exercise compared to the control group. According to the authors of the cited study, vibration therapy and massage are equally useful in preventing DOMS. In our research, vibration therapy was performed during the rest period after prolonged exercise, in contrast to the therapies being performed before exercise in the above-cited study. Despite this difference, it also observed lower Mb concentrations and lower CK and LDH activity compared to the group without vibration treatment.

Vibration applied after exercise, like in our study, was the subject of individual works [13,36]. In our previous research [36], using cycloidal vibrations with variable parameters of intensity, amplitude, and frequency after intense physical exertion in men produced a significant reduction in the concentration of Mb measured at 1 h and 24 h after exercise compared to the control group. These studies also assessed the level of metalloproteinase-2, an enzyme involved in the remodeling of the extracellular matrix, in which the concentration decreased in the vibrated group after exercise.

Currently, many devices use vibratory stimuli, most often with constant parameters describing the generated vibrations. Their impact on the body is multifaceted. Vasodilation is observed, resulting in improved blood and lymph circulation [11], reduced pain after intense exercise, and lowered levels of biochemical markers indicating damage to muscle cells [8,27]. A systematic review, published in 2019, included 10 studies with a total of 258 participants [8]. The authors of the meta-analysis reported that vibration significantly improved not only the subjective symptoms of DOMS as assessed by the VAS scale at several time points after exercise (24, 48, and 72) but also significantly improved CK levels after 24 and 48 h, but not 72 h. Our study also indicated significantly lower CK levels in the groups subjected to vibrotherapy after 24 h, which is in line with the previously observed results.

The studies cited above have confirmed vibration to reduce muscle soreness after exercise and improve the status of markers indicating the severity of inflammation and ultrastructural muscle damage. However, comparing studies involving vibrotherapy can be difficult due to the different vibratory parameters used in pre-exercise or post-exercise protocols. These parameters have a huge impact on the biological effects of treatments. The influence of vibration using both high and low frequencies on the autonomic functions of the cardiovascular system was investigated by Liu et al. [40]. The impact of vibrations on post-exercise changes in heart rate variability and peripheral arterial tension was assessed. The subjects received vibration treatments (0, 5, and 15 Hz) in random order for 10 min. The study confirmed that low-frequency vibration applied after exercise can reduce peripheral vascular tone and accelerate the recovery of the pre-paroxysmal heart rhythm. This effect was more pronounced at 15 Hz than at 5 Hz. The authors indicated these effects are a result of a decrease in the activity of the sympathetic nerves to the heart. Vibration stimuli lead to the taking over of the heart’s activity by the parasympathetic nervous system. These results encourage the use of vibration therapy after exercise, as done in our study, thanks to its ability to amplify the effects of cardiovascular influence and acceleration of post-workout recovery.

This study shows the effect of local vibration applied after prolonged physical exertion in young, healthy men. The analysis of the literature shows the complex relationship between the sex of the study participants and the use of the vibration stimulus. In the study of Shibata et al. [41], the gender differences in subjective response to whole-body vibration (WBV) under standing posture was measured. Males and females rated the discomfort of the test stimuli including fore-and-aft, lateral, and vertical vibration. Subjective scale for discomfort caused by WBV exposure was obtained. The authors concluded that there are gender differences in subjective discomfort, and that females are subjectively more sensitive for fore-and-aft and lateral WBV exposure, especially at higher vibration magnitude. The gender differences were also reported by Sañudo et al. in knee stability in response to WBV [42]. On the other hand, this stimulus may affect men and women differently, but the effect may be directly related to differences in body composition. Investigating whether WBV’s effect on force-time characteristics is dependent on time and sex was the aim of Merrigan et al.’s study [43]. Participants performed a static quarter squat with WBV (30 Hz; 2–4 mm) and then performed the isometric mid-thigh pull. In women, the rate of force development (RFD) was moderately affected immediately post-WBV. In men, however, the effect of WBV on RFD existed 15 min after exposure. Men produced more peak force (PF) than women. All RFD bands were greater in men than in women, but relative to fat-free mass, PF, and RFD, there were no differences between women and men. This indicates that the results obtained in this study concern only males, and confirmation of such an effect in females requires additional studies with female participants.

## 5. Limitation of the Study

The use of the cross-over model allowed for obtaining interesting results, but still, the basic limitation of the project is the small number of respondents. Despite the small sample size, we observed large effect size and significance of intergroup differences. We did not calculate the sample size a-priori, and therefore, we suggest that future research on this topic should include a larger sample with high statistical power.

Another limitation is the lack of blood samples after 48 and 72 h, which may provide a more complete insight into the removal rate of the biochemical markers tested. The research presented in this paper concerned the effects of local vibration in young, physically active men, and the conclusions drawn from it also apply only to this group.

## 6. Conclusions

In summary, local cycloidal-oscillating vibration reduces the activity of CK, LDH, and concentration of Mb compared to the control group, immediately and 24 h after performing 180 min moderate intensity exercise on a cycloergometer in young, physically active men. 

The introduction of treatments using local vibration after exercise can reduce post-exercise muscle damage and shorten recovery time. Such action may allow for the development of an effective, not overburdening recovery protocol in young, physically active men.

## Figures and Tables

**Table 1:** Inclusion and exclusion criteria.

Inclusion Criteria	Exclusion Criteria
- Male gender - Age 19–23 years - BMI within the range of valid values - No additional physical activity, apart from the physical activity covered by the study program - No previous athletic experience	Contraindications to participation in vibrotherapy treatments [22]: Occurring in the past: tumors, cardiovascular diseases, thrombosis, surgical interventions involving the musculoskeletal system, acute back pain, advanced diabetes; Currently present: pacemaker, acute inflammation, recovery period after endoprosthesis of the hip or knee joint, infectious diseases; Occurring during or after the treatment: headache or dizziness, nausea.

**Table 2:** Maximal values of variables measured in graded test.

Variable	Mean	SD	Min	Max
WLmax [W]	273.6	33.2	230	330
VO_2_peak [L·min^−1^]	3.28	0.63	2.27	4.22
VO_2_peak [mL·kg^−1^·min^−1^]	43.2	7.8	31.5	58.5
HRmax [bpm]	185.36	8.19	167	195
VEmax [L·min^−1^]	131.5	36.3	75.4	174.9
FRmax [breaths·min^−1^]	53.4	9.6	36	67
TVmax [L]	2.60	0.52	1.86	3.49

WLmax—maximal workload; VO_2_peak—peak oxygen uptake; HRmax—maximal heart rate; VEmax—maximal ventilation; FRmax—maximal breathing frequency; TVmax—maximal tidal volume.

**Table 3:** The concentration of myoglobin (Mb), activity of creatine kinase (CK), and lactate dehydrogenase (LDH) before (0), immediately after the renewal procedure (I), and 24 h (II) after the end of the exercise test in the group subjected to post-exercise therapy (V) and in the control group (C).

Variable	Group/*p*-Value	0	I	II
Mb (µg/L)	C	14.84 ± 4.21	32.32 ± 19.21 **	21.67 ± 8.48 **
	V	15.22 ± 4.31	23.82 ± 14.88 **	14.28 ± 5.51
	*p* (d)	0.506	0.005 * (0.44)	0.002 * (1.06)
CK (IU/L)	C	213.53 ± 34.49	306.04 ± 37.28 **	611.11 ± 55.27 **
	V	205.06 ± 25.52	270.89 ± 37.17 **	564.95 ± 41.08 **
	*p* (d)	0.501	0.030 * (0.94)	0.029 * (0.96)
LDH (IU/L)	C	172.23 ± 14.31	214.94 ± 13.92 **	210.48 ± 14.82 **
	V	174.64 ± 13.09	197.66 ± 13.80 **	194.14 ± 14.02
	*p* (d)	0.691	0.005 * (1.25)	0.014 * (1.13)

* significant differences (*p* < 0.05) between groups: C and V (post hoc); ** significant group difference to baseline (post hoc); d—effect size (d-Cohen).

**Table 4:** Differences in the concentration of myoglobin (Mb), activity of creatine kinase (CK), and lactate dehydrogenase (LDH) in the blood 1 h and 24 h after exercise in the group subjected to post-exercise therapy (V) and in the control group (C).

Variable	Group	ΔI—0	ΔII—0	ΔII—I
ΔMb	C	17.48 ± 15.61	6.83 ± 5.51 **	−10.65 ± 11.16 **
	V	8.59 ± 11.71	−0.94 ± 2.73 **	−9.53 ± 10.71 **
	*p*	0.003 *	0.000 *	0.729
ΔCK	C	92.51 ± 18.34	397.57 ± 21.33 **	305.06 ± 21.33 **
	V	65.80 ± 19.76	356.89 ± 24.61 **	294.09 ± 23.67 **
	*p*	0.002 *	0.003 *	0.245
ΔLDH	C	42.70 ± 12.00	38.24 ± 12.87 **	−4.46 ± 2.90 **
	V	23.01 ± 8.85	19.50 ± 11.76 **	−3.51 ± 7.51 **
	*p*	0.000 *	0.001 *	0.707

* significant differences (*p* < 0.05) between groups: C and V; ** significant group differences at three time points.

## Data Availability

The data presented in this study are available on request from the corresponding author.
